# Polarization-Independent Metasurface Lens Based on Binary Phase Fresnel Zone Plate

**DOI:** 10.3390/nano10081467

**Published:** 2020-07-27

**Authors:** Xing Li, Jing Tang, Jonathan Baine

**Affiliations:** 1 Shandong Provincial Engineering and Technical Center of Light Manipulations & Shandong Provincial Key Laboratory of Optics and Photonic Device, College of Physics and Electronics, Shandong Normal University, Jinan 250014, China; tjjsdnu@126.com; 2 Department of Chemistry, University of Nebraska, Lincoln, NE 68588, USA; gijoedude89@hotmail.com

**Keywords:** metasurface, lens, polarization, Fresnel zone plate

## Abstract

Based on the binary phase Fresnel zone plate (FZP), a polarization-independent metasurface lens that is able to focus incident light with any polarization state, including circular, linear, and elliptical polarizations, has been proposed and investigated. We demonstrate that the metasurface lens consisting of metal subwavelength slits can operate in a wide bandwidth in the visible range, and has a higher focusing efficiency than that of an amplitude FZP lens without phase modulation. A multi-focus FZP metasurface lens has also been designed and investigated. The proposed lens can provide potential applications in integrated nanophotonic devices without polarization limitations.

## 1. Introduction

To scale-down the size and volume of traditional optical elements, a metasurface is proposed and has been extensively studied by researchers [1,2,3,4]. A metasurface is an artificially designed 2D structure that is made up of arrays of metallic or dielectric subwavelength antennas [5,6,7,8]. The amplitude, phase, and polarization of the transmitted light can be controlled by varying the geometric parameters (width, length, or orientation angle) of the antenna [1,9,10,11]. In addition, the wavefront of the transmitted wave can be further manipulated in the desired manner by designing the arrangement of the antennas. Various ultrathin metasurface devices, such as lenses [4,9,12,13,14,15], vortex phase plates [16,17,18,19,20,21,22], holographic plates [6,23,24,25,26], and refraction deflectors [1,27,28], have been demonstrated. Lenses are fundamental optical components and play important roles in practical applications. Recently, a metasurface constructed with various meta-atoms such as V-shaped [4], rod-shaped [9,29,30,31], and C-shaped resonators [10,12] has been used to design metasurface lenses, though many kinds of metalenses have been successfully realized for micro/nano-photonic applications based on the metasurface. However, unlike traditional optical lenses, most of the metasurface lenses are sensitive to the polarization of the illumination wave [4,9,29,30,32,33]. With subwavelength rod antennas, a dual-polarity plasmonic metasurface lens that can be a convex or concave lens for left-circularly polarized (LCP) light or right-circularly polarized (RCP) light has been demonstrated [9,29]. A metasurface lens consisting of V-shaped antennas has also been achieved for only a fixed linear polarization [4]. Based on the spin–orbit interaction originating from the Pancharatnam–Berry (PB) phase, a spin-selected lens that can focus photons with different spin states to two separated focal spots has been realized in the terahertz frequency range [33]. Overall, the performance of the metasurface lens depends heavily on the polarization of incident light in most cases.

In this paper, we have proposed a metasurface lens that is capable of eliminating the dependence of the focusing property on the polarization of incident light. The metasurface consists of metal subwavelength slits that can achieve phase modulation by changing the orientation angle. Based on the binary phase Fresnel zone plate (FZP), we design the distribution of slit antennas and demonstrate the polarization-independent focusing. Moreover, the proposed metasurface lens can operate in a wide bandwidth in the visible range, and its dispersion characteristic is studied. Finally, a multi-focus metasurface lens is realized based on the proposed method. In comparison to the traditional amplitude FZP, our method shows a much higher focusing efficiency.

**Figure 1:**
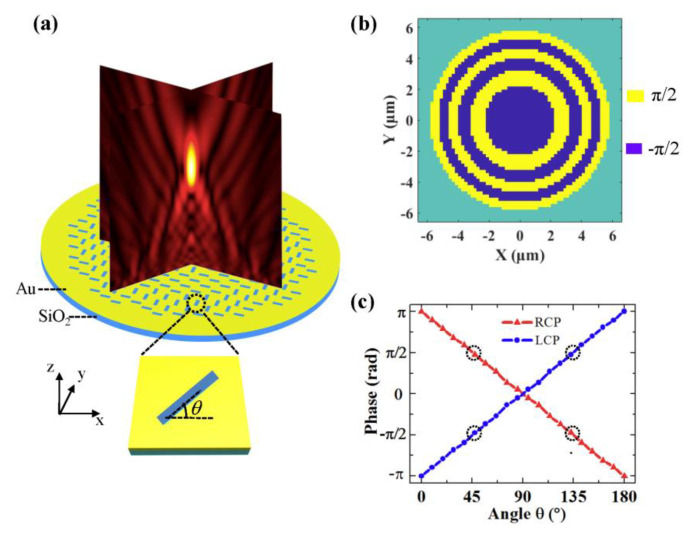
Schematic diagram of the Fresnel zone plate (FZP) metasurface lens designed for polarization-independent focusing (**a**), where the inset is a metallic rectangular slit. (**b**) Phase distributions of the binary phase FZP on its initial plane with f=8μm for λ=633nm. (**c**) Phase modulation realized by changing the orientation angles of the slits for left-circularly polarized (LCP) (blue) and right-circularly polarized (RCP) (red) light.

To realize the polarization-independent focusing, a binary phase Fresnel zone plate (FZP) metasurface lens is designed. As schematically shown in Figure 1a, the FZP metasurface consists of rectangular metallic slit antennas with different rotation angles. The slits are etched on a 100 nm Au film and the substrate is SiO_2_. A basic unit of the FZP metasurface lens is given in the inset of Figure 1a. The width and length of the slit are *w* = 50 nm and *l* = 180 nm, respectively. The orientation angle *θ* of the slit can be changed to create the required phase of the FZP metasurface lens. The required binary phase distribution on the initial plane of the FZP is shown in Figure 1b. The boundaries of the FZP can be estimated with a classical equation:

(1) $$r_{m}=\sqrt{m\lambda f+\frac{m^{2}\lambda^{2}}{4}}$$

where $r_{m}$ is the radius of the *m*-th zone boundary, λ is the wavelength of incident light, and *f* is the focal length. To make the light emerging from the FZP metasurface constructively interfere at the focal point, the light transmitted from adjacent zones should have a relative phase shift ±π. The phase of the cross-polarized wave generated by the slit antennas is calculated using commercial software FDTD Solutions. Figure 1c shows the phase profiles of the transmitted light as a function of the orientation angle *θ* for the left-circularly polarized (LCP) light and right-circularly polarized (RCP) incident light. It can be seen that the phase modulation follows the function $\Phi =2\sigma^{\pm }\theta$, where σ=±1 represents LCP light and RCP incident light, respectively [9,33]. Here, to satisfy the ±π phase shift, we choose two slit antennas with rotation angles 45° and 135° as the building block of the metasurface. The phase delays for θ=45° and θ=135° of slits are –*π*/2 and *π*/2, respectively, for LCP light, and are opposite for RCP light, as indicated by the black dotted circles in Figure 1c. Therefore, by arranging the slits with θ=45° and θ=135° in the odd zones and even zones of the FZP, respectively, we can obtain the binary phase distribution of the FZP. As the *π* phase difference between θ=45° and θ=135° slits for LCP light is equivalent to the –π phase difference for RCP light, the required binary phase distribution of the FZP can be realized simultaneously for both the LCP and RCP incident light. Considering that an arbitrarily polarized light can be regarded as the combination of LCP and RCP light, the transmitted wave can be focused by the metasurface lens as well. Then, the polarization-independent FZP metasurface lens can be achieved.

**Figure 2:**
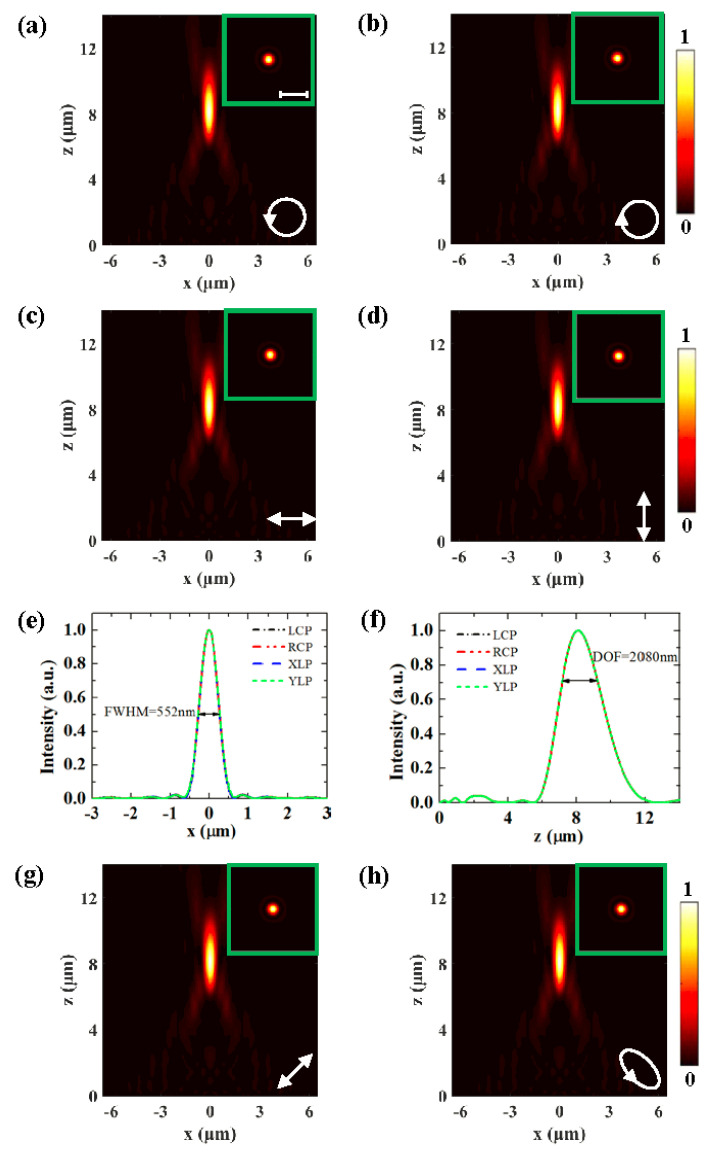
(**a**–**d**) Simulated intensity distributions at the *x*–*z* plane under LCP, RCP, *x*-linearly polarized (XLP), and *y*-linearly polarized (YLP) incidence, respectively, and the inset shows the intensity distribution in the *x*–*y* plane at *z* = 8.1 μm. The scale bar is 2 μm. (**e**) and (**f**) Transversal and longitudinal intensity profiles through the focus. (**g**) and (**h**) Intensity distributions generated by linearly polarized light (45°) or elliptically polarized incident light.

Based on the simulation with FDTD Solutions, the focusing properties of the FZP metasurface lens proposed in Figure 1 are studied. In the simulation, the diameter of the FZP is D=11.6μm, and the focal length is designed to be f=8μm. The metasurface is located at the plane *z* = 0. The period of slit antennas is set as 200 nm to avoid coupling between the adjacent slits. The dielectric constant of gold is ε=−4.723+2.388i and the permittivity of the SiO_2_ substrate is taken as 2.133. In the *x*, *y*, and *z* directions, perfectly matched layer boundaries are added to absorb the scattered light. The plane wave with wavelength 633 nm illuminates the sample from the glass substrate side. Figure 2a,b show the intensity distributions at the *x*–*z* plane of the metasurface under the incidence of LCP and RCP light, respectively. The inset is the intensity distribution in the *x*–*y* plane at the focal plane (*z* = 8 μm). We observe that a clear focal spot appears at z = 8.09 μm in the two cases, which is basically in accordance with the designed position z = 8 μm. The slight difference is caused by the limited number of slit antennas and the simulation accuracy, which restricts the FZP from ideal performance. The intensity distributions in the focal plane further verify that the transmitted fields are indeed focused into a point by the sphere metasurface lens. Under the incidence of *x*-linearly polarized (XLP) and *y*-linearly polarized (YLP) light, the normalized intensity distributions at the *x*–*z* plane are shown in Figure 2c,d, respectively. We see that the metasurface also converges the transmitted wave into a focus at *z* = 8.09 μm, because the linearly polarized light is the combination of LCP and RCP light. The results indicate the polarization-independence feature of the designed FZP metasurface lens. For a quantitative analysis of the focusing effect, the intensity profiles along the lines x=0 and y=0 through the focus are shown in Figure 2e,f. As can be seen from the figures, the curves match each other exactly, which further prove the polarization-independent focusing feature. The full-width at half-maximum (FWHM) of the focus spot is 552 nm, less than the incident wavelength of 633 nm, and the depth of focus (DOF) is approximately equal to 2080 nm. For arbitrary linearly polarized light (45°) or elliptically polarized incident light, the normalized intensity distributions in Figure 2g,h show that the transmitted light is both focused at the desired position *z* = 8.09 μm. 

**Figure 3:**
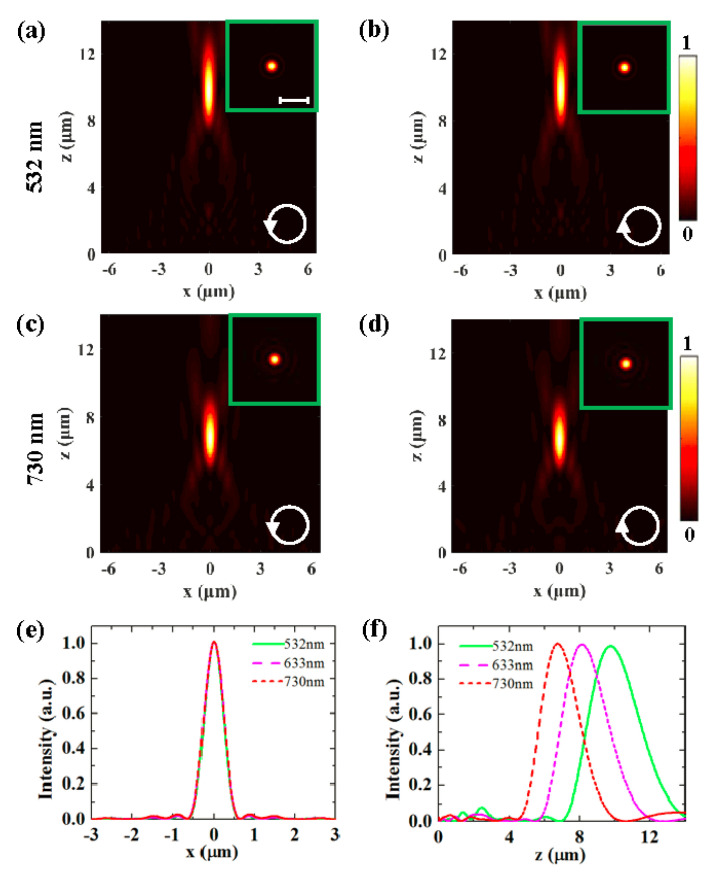
Intensity distributions for 532 (**a**,**b**) and 730 nm (**c**,**d**) incident light. (**e**) and (**f**) Comparison of the intensity profiles along the *x*-axis and along the *z*-axis for different incident wavelengths.

The frequency response of the FZP metasurface lens is analyzed by illuminating the FZP metasurface lens with two different wavelengths of 532 and 730 nm. The normalized intensity distributions in Figure 3a–d show that the focusing is still polarization-independent but the focal lengths change slightly for these two wavelengths. The intensity profiles along the *x*-axis at the focal planes are presented in Figure 3e, and the intensity profile curve for 633 nm is also presented for comparison. At these three wavelengths, the FWHMs of the focus spot remain almost unchanged. Therefore, we conclude that the proposed lens could operate in a broadband manner with good focusing performance. The intensity profiles along the *z*-axis for different wavelengths are shown in Figure 3f. The focal length decreases with the increase in the incident light wavelength. The corresponding focal lengths for the 532 and 730 nm incident light are 9.7 and 6.7 μm, respectively.

**Figure 4:**
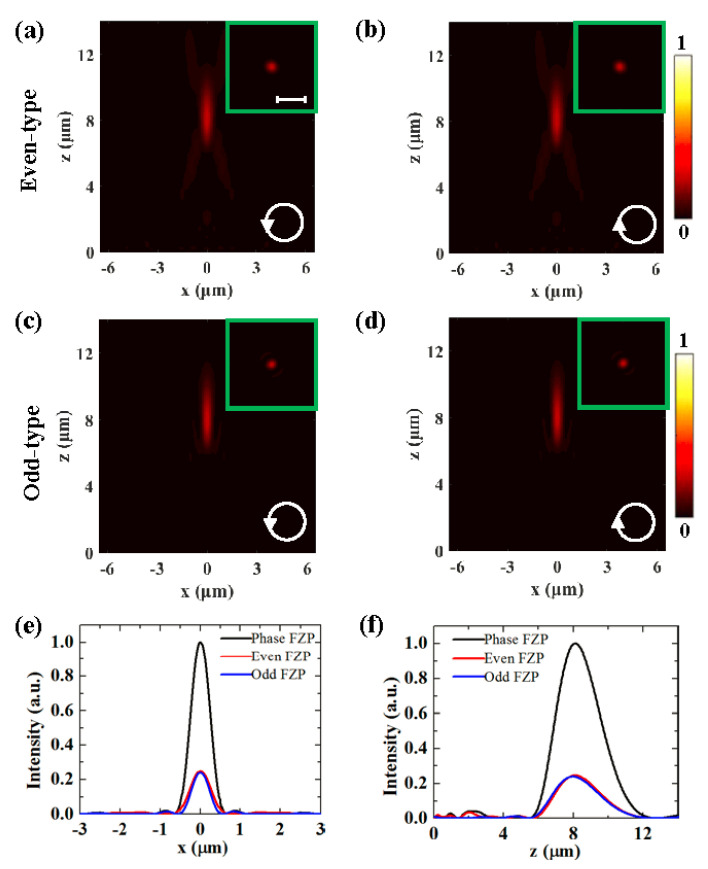
Intensity distributions generated by the even-type (**a**,**b**) and odd-type FZP (**c**,**d**), respectively, under LCP (**a**,**c**) and RCP (**b**,**d**) incidence. The corresponding normalized intensity profiles along the *x*-axis (**e**), and along the *z*-direction (**f**).

There are a few previous studies that have investigated the polarization-independent metasurface lens made up of subwavelength holes or rings. The phase of the transmitted wave or reflection wave can be manipulated by varying the diameters of the holes or rings [34,35]. As the isotropic characteristic of the hole or ring, they are insensitive to the polarization of incident light. It is no wonder that the metasurfaces can realize polarization-independent focusing by combining such holes or rings to achieve the desired phase profiles. Our design is based on the rectangular slit, which is anisotropic, and the phase of the transmitted light depends on the incident polarization. In contrast to the slit metasurface lens utilizing the phase profile of the traditional sphere lens [34], this design is able to realize polarization-independent focusing due to the benefit of binary phase modulation. Without the binary phase modulation, the slit antennas are distributed in either odd or even zones, which is in analogy with the traditional amplitude FZP. The amplitude FZP lens has the same radius, focal length, and geometry of slit antennas as the binary phase FZP metasurface lens. Figure 4a–d show the simulated intensity distributions generated by the even- type and odd-type FZP, respectively, under LCP and RCP incidence. The corresponding normalized intensity profiles along the *z*-axis and along the *x*-direction are compared in Figure 4e,f. It is noted that the peak intensity of the amplitude FZP lens is about one quarter of that of the binary phase FZP metasurface lens, which is due to the fact that only half-zone nanoslits constructively interfere at the focus. We have also calculated the focusing efficiency by defining it as the ratio of the power integration of the focus (with a radius of just three times the FWHM spot size) to the incident power [36]. For the binary phase FZP metasurface lens, the focusing efficiency is calculated to be 4.1%. For the even- type and odd-type FZP, the focusing efficiency is both 1.1%. Therefore, we concluded that the binary phase FZP metasurface lens considered here has a higher focusing efficiency (about 4 times) than the amplitude FZP lens, as shown in Figure 4. The proposed FZP metasurface lens has a much smaller volume than the traditional optical lenses and can eliminate the dependence of focus on the polarization of incident light. The binary phase modulation method avoids complex constraints of design. The metasurface lens can operate in a wide bandwidth in the visible range. However, the focusing efficiency of the metasurface is lower than those of traditional optical lenses. The efficiency could be significantly improved by enabling the lens to work in the reflecting mode [6]. Thus, in principle, the efficiency of the metasurface lens can be improved to the level of practical applications.

**Figure 5:**
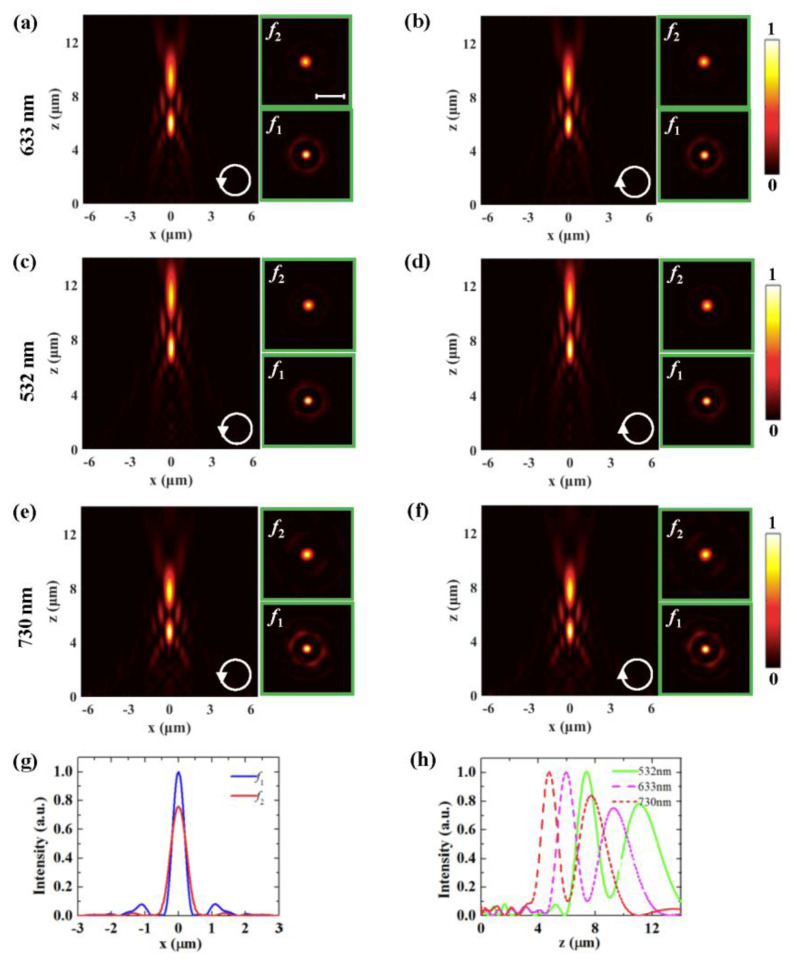
Intensity distributions generated by multi-focus FZP metasurface lens for LCP (**a**,**c**,**e**) and RCP incident light (**b**,**d**,**f**) at 633 (**a**,**b**), 532 (**c**,**d**), and 730 nm (**e**,**f**). The upper and lower panels show the intensity distributions at two focal planes. The transversal (**g**) and longitudinal (**h**) intensity profiles in the corresponding focal planes.

Moreover, based on the proposed method, the multi-focus FZP metasurface lens can be realized. The metasurface is divided into two regions, with one area corresponding to focal length *f*_1_ and the other corresponding to focal length *f*_2_. The two types of nanoslit arrays are alternately arranged in the designed region. A multi-focus FZP metasurface lens with two focal lengths, *f*_1_ = 6 μm and *f*_2_ = 9 μm, is designed for λ=633nm light. Figure 5a,b show the simulated field distributions at the *x*–*z* plane for LCP light and RCP light, respectively. The upper and lower panels in Figure 5a show the intensity distributions at two focal planes. Two focuses at *z* = 5.97 and 9.22 μm are obtained for 633 nm illumination at both polarizations, which is as expected in the original design. The ratio of the peak intensity at the center of the two focal spots is about 1:0.75. Figure 5c–f show field distributions at the *x*–*z* plane for LCP light and RCP light at 532 and 730 nm, respectively. The transversal and longitudinal intensity profiles in the corresponding focal planes are presented in Figure 5g,h, respectively. We can obtain that the FWHMs are 423 and 581 nm at the two focal planes of *f*_1_ = 6 μm and *f*_2_ = 9 μm for the 633 nm incident light, respectively. The focal lengths of the multi-focus lens decrease with the increase in the incident light wavelength. Focal points can be clearly observed for the two cases, showing little dependence on the incident polarizations. The multi-focus FZP metasurface lens has the advantages of polarization-independence and broadband performance as well.

## 2. Conclusions

In conclusion, we have demonstrated the polarization-independent metasurface lens based on the binary phase FZP. The metasurface lens can focus the incident light with any polarization state. The design of the lens is based on the phase modulation of the metal subwavelength slits. In comparison to the traditional amplitude FZP, our proposed metasurface lens has a much higher (about 4 times) focusing efficiency. Moreover, the metasurface lens can operate in a wide bandwidth in the visible range. In addition, a multi-focus FZP metasurface lens is realized. The method and the results in this paper may play important roles in designing polarization-independent metasurface devices.
